# Primary Mediastinal Large B-Cell Lymphoma As an Incidental Finding: Report of a Case

**DOI:** 10.4274/tjh.2016.0057

**Published:** 2018-05-25

**Authors:** İpek Yönal-Hindilerden, Fehmi Hindilerden, Serkan Arslan, İbrahim Öner Doğan, Meliha Nalçacı

**Affiliations:** 1 İstanbul University İstanbul Faculty of Medicine, Department of Internal Medicine, Division of Hematology, İstanbul, Turkey; 2 Dr. Sadi Konuk Training and Research Hospital, Clinic of Hematology, İstanbul, Turkey; 3 Dr. Sadi Konuk Training and Research Hospital, Clinic of Radiology, İstanbul, Turkey; 4 İstanbul University İstanbul Faculty of Medicine, Department of Pathology, İstanbul, Turkey

**Keywords:** Mediastinal neoplasm, B-cell lymphoma, PMBCL

**To the Editor,**

A 21-year-old female was examined for an incidentally detected left parahilar mass on chest radiograph which was taken at the time of job application (Figure 1a). Thoracic computed tomography revealed a mass of 10x9x5 cm with irregular lobulated borders in the anterior mediastinum invading the pericardium (Figure 1b). Histopathological examination of the anterior mediastinotomy material revealed large neoplastic B cells staining positive for CD20 and MUM-1, negative for CD10, and with a high Ki-67 proliferation index (80%-90%) (Figure 2). On positron-emission tomography scan, only the mediastinal mass showed increased fludeoxyglucose uptake (SUV_max_: 18) (Figure 1c). Final diagnosis was stage 1A primary mediastinal large B-cell lymphoma (PMBCL). After 6 cycles of R-CHOP, PET scan showed partial anatomical and metabolic response. R-CHOP was completed to 8 cycles followed by mediastinal radiation. She has now been disease-free for 2 years.  

PMBCL, accounting for 2%-4% of all non-Hodgkin lymphomas, often presents as a bulky anterior mediastinal mass and often invades surrounding structures such as the heart, lungs, pleura, and superior vena cava [1,2]. Patients often present with cough, dyspnea, chest pain, and superior vena cava syndrome [3]. R-CHOP plus consolidative mediastinal radiation is often an option [4]. Herein, we report a rare case of asymptomatic PMBCL with bulky mediastinal mass in which the patient achieved complete remission after R-CHOP and mediastinal radiation.

## Figures and Tables

**Figure 1:**
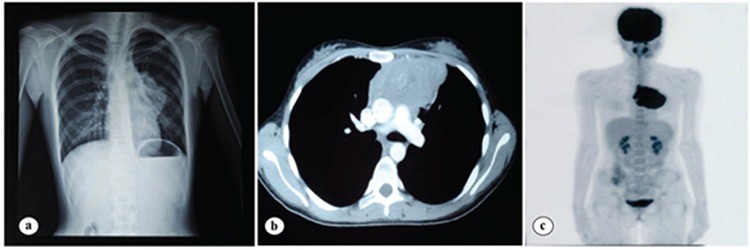
Radiological findings of primary mediastinal B-cell lymphoma. a) Appearance of the left parahilar mass on chest plain film. b) Thorax computed tomography depicts a mass of 10x9x5 cm in the anterior mediastinum with irregular lobulated borders invading the pericardium. c) Positron-emission tomography scan shows increased fludeoxyglucose uptake in the tumor.

**Figure 2:**
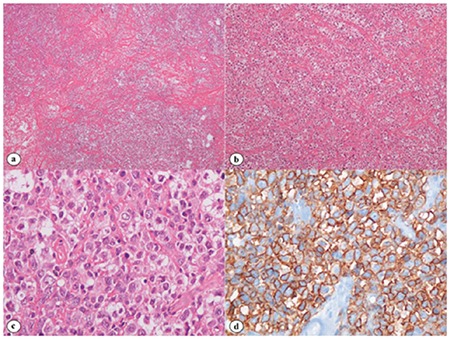
Histopathological examination of the mass. a) Diffuse neoplastic infiltration on a partially sclerotic background (hematoxylin and eosin stain, 40^x^). b) The clear-cell appearance of the tumor cells (hematoxylin and eosin stain, 100^x^). c) The appearance of round nuclei (centroblast-like) and clear cytoplasm (hematoxylin and eosin stain, 400^x^). d) Infiltrated cells with CD20 expression (hematoxylin and eosin stain, 400^x^).
